# Understanding the mental health needs of Scotland's prison population: a health needs assessment

**DOI:** 10.3389/fpsyt.2023.1119228

**Published:** 2023-05-17

**Authors:** Lindsey Gilling McIntosh, Cheryl Rees, Caroline Kelly, Sheila Howitt, Lindsay D. G. Thomson

**Affiliations:** ^1^Centre for Clinical Brain Sciences, Division of Psychiatry, University of Edinburgh, Edinburgh, United Kingdom; ^2^Forensic Mental Health Services Managed Care Network, Carstairs, United Kingdom; ^3^Department of Forensic Psychiatry, The State Hospitals Board for Scotland, Carstairs, United Kingdom

**Keywords:** mental health, vulnerable population, Scotland, health needs assessment, prison healthcare

## Abstract

**Introduction:**

This study reports on an assessment of mental health needs among Scotland's prison population which aimed to describe the scale and nature of need as well as identify opportunities to improve upon the services available. The project was commissioned by the Scottish Government to ensure that future changes to the services available to support the mental health and wellbeing of people in prison would be evidence-based and person-centered.

**Methods:**

A standardized approach to health needs assessments was employed. The study was comprised of four phases. In phase I a rapid literature review was undertaken to gather evidence on the prevalence of mental health needs experienced by people in prison in the UK. In Phase II a multi-method and multi-informant national mapping exercise involving providers to all Scottish prisons was undertaken to describe the mental health services available, and any gaps in these services, for people in and leaving prison. In Phase III prevalence estimates of several mental health needs were derived for Scotland's current prison population, modeled from a national survey dataset of Scotland's community population using logistic regression. Finally in Phase IV, professional stakeholders and individuals with lived experience were interviewed to understand their experiences and insights on challenges to supporting the mental health and wellbeing of people in prison, and ideas on how these challenges could be overcome.

**Results:**

Evidence across the four phases of this needs assessment converged indicating that existing provision to support the mental health of people in prison in Scotland was considered inadequate to meet these needs. Barriers to effective partnership working for justice, health, social work and third sector providers appear to have led to inadequate and fragmented care, leaving prisoners without the support they need during and immediately following imprisonment.

**Conclusions:**

Joint and coordinated action from justice, health and social care, and third sector providers is needed to overcome enduring and structural challenges to supporting the mental health of people in prison. Eighteen evidence-based recommendations were proposed to the Scottish Government relating to the high-level and operational-level changes required to adequately meet the prison population's mental health needs.

## 1. Introduction

People in prison are more likely to have mental health needs^1^ than the general population, from common problems such as anxiety, depression and substance dependency to serious mental disorder including schizophrenia (1, 2). These mental health needs are highly comorbid, meaning these individuals frequently experience multiple co-occurring problems (3). For many, these issues precede imprisonment and are thought to be associated with predisposing factors such as higher rates of traumatic or adverse life experiences (4) and head injury (5). Individuals who come into prison are also more likely to be from communities characterized by multiple deprivation (6), to have spent time in local authority care (7), and to have experienced interpersonal victimization (8). Imprisonment itself, however, can also be damaging to someone's mental health, with the remand period recognized to be one of particular vulnerability (9, 10).

Prison healthcare should be informed by the principle of equivalence, and offer the same level, range, and quality of healthcare as that provided in the community (14). However, some argue that this does not go far enough and that, to compensate for the levels of deprivation, risk factors for poor mental health and health inequalities experienced by the prison population, equivalence of health *outcomes* should be the focus (15). Unfortunately, not all people in prison with mental health needs are engaged with services to address these needs. Public stigma around mental illness and distrust of health professionals lead to a reluctance to disclose ongoing problems (11, 12). Ineffective screening procedures by prison in-reach teams and underdeveloped care pathways often fail to identify and meet mental health needs among this population (13, 16). However, the scale of need and degree of comorbidities often far outstrip the resources available to support individuals even when their needs are known. For example, a survey of prisons in England and Wales found mental health staffing numbers falling well short of equivalence with community mental health services and noted striking variation in mental health staffing between prisons, which has been likened to a “postcode lottery” within prison mental healthcare (14).

The literature stresses the importance of systematic and collaborative approaches to care pathways for people in prison who have mental disorder (17). There are five primary elements of effective service provision across the prison care pathway that have emerged from this literature. These are described in the STAIR model, an acronym that stands for screening, triage, assessment, intervention, and re-integration (18). The STAIR model is a framework that defines and measures prison mental health services as a clinical pathway with a series of measurable and linked functions (17). This should include, for all people coming into prison, an initial screen from trained mental health staff using validated tools to identify presenting issues which require immediate intervention (psychosis, suicidality, and substance withdrawal), followed by a subsequent second screen which provides a more detailed assessment of the individual's mental health need and current functioning. Individuals should then be triaged to the appropriate service and level of care following multidisciplinary case discussion of the information derived from screening. Research shows that ~15% of the prison population (2) will require assessment by specialist mental health services at this stage. Then, a range of culturally competent and evidence-based interventions should be available, tailored to the severity of the individual's needs. Finally, planning for community reintegration, with specification of the appropriate package of care for the individual transitioning to the community, should begin well in advance of release. There is a growing literature of studies evaluating prison mental health services against these standards, though more work is needed on standardized assessment approaches (17). There is a clear advantage to embedding data collection processes that enable service evaluation and quality improvement within existing clinical governance procedures. The STRESS-Testing approach (19) employed within an Irish remand prison demonstrates how such data, which covered screening, identification, service caseload, transfer of care, diversions, efficiency, self-harming behaviors, and service mapping, could be studied to identify aspects of service provision requiring improvement.

Health needs assessment is systematic tool to review the health issues facing a population and the effectiveness of healthcare services currently in place. Health needs assessments are often used to inform the commissioning and planning of health services (21). In the prison context, where demand for health care often appears to outstrip the capacity of services, a health needs assessment can help prison-based health services to plan their health care provision and move toward a service which will tackle health needs systematically rather than reacting to demand (20). Health needs assessments may incorporate elements of one or more categories: survey approach, rates-under-treatment approach, social indicator approach, key informant approach, and community forum (22). They typically utilize a variety of data sources and quantify incidence and/or prevalence of various health outcomes. One central feature to health needs assessment is the differentiation of true health need, the demand for healthcare services, and the supply and availability of these services (20).

Within the UK, there have been few attempts at a national level to systematically assess the mental health needs of individuals in prison, and none recently. In 1998 the Office of National Statistics published a landmark report on the mental health needs of people in prison (23). Over 3,000 people were surveyed and assessed through standardized clinical interviews across all prisons in England and Wales. Through comparison to the general population, the study reported clear evidence of increased psychiatric morbidity among the prison population across a range of mental health problems, including major mental disorder, personality disorder, substance use and self-harm. In Scotland, two national needs assessments were also conducted in the 1990s (24, 25), followed by a comprehensive national healthcare needs assessment published by the Scottish Prison Service (SPS) in 2007 (26). Though the Graham (26) report remains the most recent national assessment of mental health needs in Scotland's prison population, it has been noted that the policy impact of its findings were limited by the report's reliance on existing data held by SPS to estimate the prevalence of mental health needs across Scottish prisons. At a local level, several NHS Scotland Health Boards have undertaken or are undertaking prison mental health needs assessments to inform planning for future service provision (27, 28).

Scotland has a prison population of 7,504 (*n* = 283 women) across 15 prisons, including one open, low supervision prison (29). SPS publicly manages all but two prisons, which are currently operated by private contractors. Accounting for its national population, Scotland's rate of imprisonment, 137 per 100,000, is one of the highest within Western Europe, alongside England and Wales. The Scottish prison population is 95% White, with the largest ethnic minority groups being Asian (2%) and African, Caribbean or Black (2%). Though the prison population size overall has been relatively stable in recent years, an increasing proportion of the prison population is on remand (pre-trial). During the year of the present needs assessment, ~25% of the prison population was on remand, which is the highest on record (29). The proportion of sentenced individuals in prison has dropped 15% since 2020, driving reductions in the proportion of women and young people under the age of 21 in prison.

SPS endorses a “whole-prison” approach (30) to health improvement, which advocates for addressing health factors through comprehensive and integrated programmes and recognizes a role for health promotion in all prison staff. While mental health is a whole-prison concern, involving multiple agencies working in partnership, NHS Scotland has been responsible for the delivery of primary and community healthcare in prisons in Scotland since 2011. Individuals in prison or who are accused of a criminal offense who have severe mental illness, or those with particularly complex needs, can access specialized, tertiary care including assessment and treatment by transfer to one of 20 high, medium, low security psychiatric units, locked wards or intensive psychiatric care units which accept transfers from prison. Secure hospitals, part of Scotland's forensic mental healthcare system, represent a largely separate system of care but one which interfaces heavily with prison mental healthcare in its operation. A range of third and voluntary sector organizations also provide programming and services, including throughcare support, to promote the mental health and wellbeing of people in prison in Scotland. Since 2011, SPS has had a more limited operational role in support for mental health services in prisons though it continues to be involved in promoting wellbeing, in identifying and supporting individuals with mental health needs in prison and in implementation of its suicide risk management strategy, “Talk to Me” (31).

Evidence has accumulated in recent years that existing prison mental health services in Scotland are not proactively designed to meet the needs of their patient groups. The provision of mental health services across the Scottish prison estate is variable and in need of improvement to meet the scale and nature of need (32, 33). There are recognized problems implementing the whole-prison health promotion approach and evidence of silo working among health, social work, SPS and third sector agencies (32, 34). The sustainability of the current mental healthcare model in prisons has been questioned, with likely demand outstripping available resources (35, 36). This is in part due to concerns about the numbers of nursing staff and the ability to provide an effective mental health service with clinical time routinely taken up by treating substance use problems (36, 37).

In 2020, the Scottish Government commissioned a series of national needs assessments in relation to Scotland's prison population to ensure that future changes to prison healthcare are person-centered and evidence based. This work culminated in the publication of four reports, on social care support (38), physical and general health (39), substance use (40), and mental health needs (41). This paper reports on the work of the mental health needs assessment, which was conducted from July 2021 to March 2022. By this time, an in-depth national mental health needs assessment was overdue, with SPS and the National Prisoner Healthcare Network calling for it in substantive reports in 2007 (26), 2014 (33), and 2016 (32). This study used a triangulation of sources and the best available data to determine the scale and nature of mental health needs within Scotland's prison population, to understand current service provision in custody, and as part of throughcare, and engage with stakeholders to gather their views and insights on current challenges.

## 2. Study procedure and governance

The study followed the Health Needs Assessment in Prisons approach (20) and incorporated three main elements of needs assessments: corporate, epidemiological, and comparative. In the corporate approach, stakeholders and others with special knowledge are engaged to determine their views on what is needed. In the epidemiological approach, the incidence and prevalence of various needs are described. Finally, in the comparative approach, existing services are compared with the services of other providers and major discrepancies are examined and address. The needs assessment was conducted in four substantive, linked phases: rapid literature review, service mapping exercise, quantitative analysis, and qualitative interviews with stakeholders. The latter three phases are reported here. Broadly, the service mapping exercise fulfilled the comparative element of health needs assessment, the quantitative analysis fulfilled the epidemiological element, and the qualitative interviews fulfilled the corporate element. Expertise and guidance was received throughout the project from a Research Advisory Group featuring representatives from health, justice, third sector, Scottish Government, and those with academic expertise in prison mental healthcare. The study also received input from a Lived Experience Panel, comprised of individuals who have previous experience of imprisonment and those who currently work to support individuals who have recent experience of being imprisoned.

The University of Edinburgh Medical School Research Ethics Committee and the NHS South East Scotland Research Ethics Committee confirmed that as a service evaluation the study was exempt from full research ethics review by their committees. The Scottish Prison Service Research Access and Ethics Committee provided access and ethical clearance to engage with SPS staff and residents, and to access data held by SPS. Face-to-face data collection and interviewing was not possible during the course of the study due to the ongoing risks and challenges relating to COVID-19. Written informed consent for participation was not required for this study in accordance with national legislation and institutional requirements. However, for good practice written informed consent was received from lived experience participants.

## 3. Service mapping exercise

### 3.1. Approach

NHS Scotland is responsible for the provision of healthcare including mental healthcare to those in prisons, but it is recognized that other partners including SPS, prison-based social work teams and third sector organizations work together and independently to support the mental health of individuals in prison. A national mental health services mapping exercise was previously conducted in 2012 by the Forensic Mental Health Services Managed Care Network on behalf of the National Prisoner Healthcare Network Mental Health Subgroup (33). That mapping exercise found that service provision in nursing and psychiatry was related to historical factors rather than a true assessment of need, and there was very little input from clinical psychology into prison mental health teams across the country.

The aim of the present service mapping exercise was to understand current provision available to people in all of Scotland's prisons. The mapping exercise was undertaken by the Forensic Network, selected for its experience in conducting the previous national mapping exercise and for its links with prison health centers in relation to the care of individuals who require transfer from prison to forensic hospitals. Electronic proformas were sent to prison health center managers and prison based-social work team leads across all 15 prisons for completion and return in September 2021. A 100% completion and return rate was achieved. To gather third sector input into the mapping exercise, the research team and the Forensic Network partnered with a network called the Criminal Justice Voluntary Sector Forum (CJVSF), which connects over 30 third sector organizations working in criminal justice settings in Scotland. Input was gathered through proforma response and from a virtual discussion event hosted by CJVSF with attendance from organizations which support the mental health of individuals in and leaving prison.

### 3.2. Service size and configuration

Integrated primary and secondary mental health services are available in 13 of the 15 prisons in Scotland, with mental health services offered only through primary care in two prisons. Mental health and substance use services were found to be integrated in six establishments. In nine prisons these services were not formally integrated though work closely and collaboratively. Service staffing, according to number of qualified or registered professionals, across nursing, allied health professionals, psychiatry, and clinical psychology as reported by health center managers is set out in Table 1. Workforce figures are reported using the local standard by discipline; namely whole time equivalent [1 whole term equivalent (WTE) = full time / 37.5 h per week] for nursing and allied health professionals (AHP), and number of sessions per week (one session = ½ day, 10 sessions per week) for psychiatry and clinical psychology professionals. Table 1 includes the workforce totals by profession as well as the median and range per establishment, using prison resident to staff ratios in order to standardize prison size. Figures are reported separately for the closed and open prison estate. Scotland's only operating open prison is HMP Castle Huntly, which has a minimal NHS mental health team consistent with SPS's approach that individuals who are acutely mentally ill or experiencing a mental health crisis would not remain in the open estate. In such instances, the individual would be transferred back to closed conditions where their needs can be more closely and safely monitored and their mental health stabilized.

**Table 1:** NHS mental health service resource in the Scottish prison estate.

	**Unit**	**Total (across estate)**	**Prison residents per staff**		
			**Closed estate**		**Open estate**
			**Median**	**Range**	
Nurses (any specialty; *k* = 15)	1 WTE	90.6	86	26–182	925
Mental health nurses (*k* = 15)	1 WTE	75.6	94	25–282	927
AHPs (*k* = 8)	1 WTE	9.6	391	237–1,817	925
Psychiatry (*k* = 15)	Half-day sessions	39	219	32–455	741
Clinical psychology (*k* = 13)	Half-day sessions	165	56.5	20–121	185

Across the prison estate there were 91 WTE nurses employed, with 76 being mental health nurses. The mental health teams in three prisons were noted to also include substance use or learning disability nurses. There was substantial variation across in the resident-to nurse ratio between prisons. The women's prison HMP YOI Cornton Vale reported one of the highest nursing staff complements. There were mental health nurse vacancies noted at six prisons, in several cases there were multiple unfilled posts within a prison. AHPs including occupational therapists and speech and language therapists formed part of the core multidisciplinary mental health teams in just over half of establishments, though there was wide variation across these prisons in terms of input per resident. Only eight of Scotland's 15 prisons employed AHPs as part of the mental health team. Across the entire prison estate there were 9.6 WTE AHPs employed, a quarter of them in one prison in eastern Scotland. It should be recognized that in prisons where AHPs were not reported to be part of the mental health team they may nevertheless provide support to individuals in the prison who have mental health needs. There was psychiatry input into each prison, totalling to 39 sessions (equivalent to just under four full time psychiatrists for the prison estate). The number of funded psychiatry sessions per week appeared relatively arbitrary^2^ in relation to prison size, with relatively few sessions in several of the largest prisons serving Glasgow and Edinburgh. Thirteen prisons had clinical psychology input, totalling to 165 sessions across the estate (over 16 full time clinical psychologists for the prison estate). Review of the resident to staff ratio for each prison across these professions yielded evidence of inequities in terms of mental health input into certain prisons, and arbitrary service resource allocation not closely linked to the number of prison residents.

### 3.3. Service delivery

#### 3.3.1. Screening and referral

All people being received into prison in Scotland complete a standardized health screening by a member of the prison nursing team, most often a general rather than mental health nurse. The mental health portion of the screening asks about previous history of mental illness, self-harm, prior contact with mental health services, previous inpatient admissions for psychiatric care and any medication prescribed at the time of reception into prison. A referral to prison mental health services can be made following screening where there is a current mental health concern or the individual is in receipt of medication for a mental health or substance use problem. Responses from three establishments recognized that the process could be more thorough, or that a mental health nurse should deliver that mental health screening. Social work and third sector colleagues highlighted the need for a more robust process in place to identify mental health needs for those coming into prison, however only one NHS team identified issues with the existing process.

#### 3.3.2. Multidisciplinary case management

There was broad consistency in approach to the multi-disciplinary case management of mental health assessment and treatment. Most establishments reported having a larger fortnightly or monthly meeting called the multidisciplinary mental health team (MDMHT) meeting. MDMHT meetings are chaired by SPS and feature wide professional representation including, typically, forensic psychology, substance use nurses, social work and prison staff in addition to representation from the NHS mental health team. Respondents stated the these meetings have several purposes, including to discuss any mental health concerns amongst the individuals within the prison establishment, to review management of individuals on the Talk to Me strategy, and to discuss potential hospital transfers. Prisons which had a dedicated mental health team reported in nearly all cases a weekly or fortnightly NHS mental health team meeting, with health professionals that comprise the core service in each establishment. Respondents reported that in these meetings, existing cases may be reviewed, relevant complex care concerns identified and access to further assessments and interventions by the mental health team are discussed. In addition to these two primary forums, respondents detailed a range of multi-disciplinary meetings convened to support individuals, at which mental health or substance use concerns are discussed where relevant, on a case-by-case basis. These include Care Programme Approach meetings for the coordination of transitional care, Talk to Me Conferences, integrated case management meetings, and risk assessment and management meetings.

#### 3.3.3. Interventions

Respondents described specific interventions delivered by members of the multidisciplinary team (MDT) offered to support individuals' mental health. Most establishments reported a range of individual and group interventions for common mental health and substance use problems, according to a tiered approach. Interventions vary in intensity and in the staff who deliver them. For example, information and self-help interventions, such as self-help pamphlets and literature and relaxation CDs, are available to individuals in prison without the need for referral. Other low intensity interventions involve direct clinical contact, initiated usually by clinical psychology, though they are facilitated or co-facilitated by nursing staff and other non-health colleagues, including prison based social work and SPS staff in certain establishments. These low intensity interventions typically target common and less severe mental health problems, for example anxiety management, mindfulness, psychoeducation and coping skills. High intensity interventions are typically delivered by clinical psychology and can include cognitive behavioral therapy, acceptance and commitment therapy, and trauma therapy. Interventions for personality disorder are delivered by clinical psychology, are driven by the individual's case formulation, and span a range of therapeutic models including cognitive analytic therapy, schema therapy, mentalization-based therapy and cognitive behavioral therapy. No establishments described a specific service or intervention in place for the prevention of suicide other than the local implementation of the Talk to Me strategy. There was little evidence of differential access to interventions for certain groups of individuals (for example, by the individual's gender or legal status) within prison, except for psychological interventions which in many cases is not initiated for individuals with <6 months to serve before their earliest date of liberation (release from prison).

#### 3.3.4. Discharge planning and throughcare

Discharge planning and throughcare generally followed a matched-care approach whereby a referral is made to the relevant community mental health team if ongoing support will be required following liberation from prison, for example in cases where the individual is receiving antipsychotic medication or would benefit from further psychological intervention. On a case-by-case basis, case conferences are held to plan for the transition of care, to which community providers are sometimes invited. Social work teams described a significant role for their profession in liaising with community agencies and third sector services on behalf the wider MDT. If ongoing support following liberation is not considered required, the individual is provided with information and advice on community mental health services and signposted to their general practitioner (GP) as the first point of contact for any developing problems. Individuals on medication-assisted treatment for substance use problems are provided with an appointment to attend the community substance use team on the day of, or the day following, liberation.

### 3.4. Issues and challenges

Professionals involved in the mapping exercise were asked to comment on whether there were service gaps or other barriers beyond mental health service provision, to meeting the mental health needs of individuals in their establishment.

#### 3.4.1. Funding and service provision

Responses received from 12 NHS and seven social work teams recognized that the mental health needs of individuals in prison appeared to outstrip current mental health service resources. As a result, mental health teams must direct most of their resources to a relatively small proportion of the prison population who are acutely unwell, acknowledging that there are many more who have less severe, or less complex needs which would benefit from care but who are not “unwell enough” to progress past long waiting lists. Individuals in the community with mild or transient mental health problems would more easily be able to seek out and access self-help materials and digital health interventions, whereas these options are limited in prison. Two prison-based social work teams also highlighted inadequate funding to their service, citing that this limited their ability to support individuals on their caseloads with mental health needs.

#### 3.4.2. Staffing

Respondents noted that staffing deficits, which existed prior to the pandemic, were exacerbated by COVID-19-related sickness absence and self-isolation requirements. Due to staff shortages during the pandemic, mental health nurses were required to cover shifts in the wider health team. This resulted in the cancellation of clinics and assessments or reviews of individuals in prison. Mental health nurses being pulled from their duties away to support wider health services was also an issue prior to the pandemic. Distinct from COVID-19 related issues several mental health teams highlighted difficulties in recruiting staff to posts, primarily mental health nurses.

#### 3.4.3. Substances

Responses reflect the considerable challenge for mental health service provision from issues relating to access to and use of substances within prison, and the high proportion of people in prison who have dual diagnoses. NHS teams reported that changes in patterns and prevalence of substance use was driving mental health referrals, for example an increase over a 12-month period in the use of novel psychoactive substances was considered to be driving an increase in referrals related to drug-induced psychosis. Please note that a separate needs assessment into substance use needs (21) was commissioned by Scottish Government which explored this issue in detail.

#### 3.4.4. Sharing information

NHS and social work teams both highlighted difficulty accessing relevant health information on individuals in prison. The experience of information sharing and handover between services based in the community and in prison was highlighted as poor in many cases, describing delays and the need for attempts to chase up reports retrospectively. Social workers highlighted frustrations regarding barriers to non-health staff accessing information from their health colleagues, reporting that as a result social work is sometimes required to complete risk assessment and management tools with limited or inaccurate information relating to an individual's mental health. For national service prisons such as HMP YOI Cornton Vale and HMYOI Polmont, which receive individuals in prison from a number of NHS Boards, accessing prior health records from other NHS Boards and held on other clinical information systems was reported to be difficult and time consuming.

#### 3.4.5. Non-English speakers

Several social work teams described barriers to accessing prison mental health services linked to residents whose first language is not English. They described difficulty accessing translators for some appointments.

#### 3.4.6. Partnership working

Challenges in effective partnership working was a recurrent theme raised in relation to barriers to meeting the mental health needs of individuals in prison. Three social work teams suggested that an increased awareness of the roles and responsibilities of all professionals involved in care of people in prison would better facilitate joint working. This was also highlighted by representatives from third sector organizations, who reported difficulties getting access into prisons to deliver services due to the inflexible structures in place, and also an under-recognition by NHS and statutory colleagues of the value of non-clinical services offered by third sector organizations.

#### 3.4.7. Transitions

Service providers highlighted the impact that the process of transitioning from prison to the community can have on someone's mental health is under-recognized. Upon liberation, people often return to similar circumstances in the community as they were in before prison, and which may have been made worse by or during imprisonment. Several respondents indicated that current support for employment, housing, and existing pre-release planning and throughcare support for mental health and substance use (limited largely to referral to community services) is inadequate and sets the individual up to fail upon release.

#### 3.4.8. Facilities and prison regime

NHS teams in three prisons indicated that limited available physical space within the establishment for clinical and office spaces was an operational challenge. This was worsened at times during requirements for people to maintain a necessary minimum physical distance due to the pandemic. Multiple services highlighted that the limited window of 2 h available each morning and afternoon for health center clinics was problematic on account of working within the time constraints of the SPS regime (e.g., requirement of prison staff escort to health center, closure of health center at 5 pm).

#### 3.4.9. Training

NHS teams reported good availability of training relevant to mental health through a range of sources including their local NHS Board, NHS Education for Scotland, and the Forensic Network's School of Forensic Mental Health. Social work teams overwhelmingly stated that they would welcome funding for and access to training related to mental health. Responses indicated there was no mandatory training relating to mental health (with the exception of training on the Talk to Me programme), despite the recognized high prevalence of mental health needs among people in prison. Social work teams viewed a foundation level of training on mental health as integral to good risk assessment and management planning. There was consistent recognition that some level of mental health training should be mandatory for all staff working in prisons including and in particular, prison staff as this staff group spend the most time with people in prison.

#### 3.4.10. COVID-19

The pandemic was noted to have exacerbated many of the pre-existing challenges in service delivery. It also strained MDT working (through reliance on video conferencing and physical distancing requirements affecting team meetings). In prisons within NHS Boards where access to Near Me was limited, direct patient therapeutic activity ceased for a prolonged period during the pandemic.

With these challenges however, have also come positive learning points. Several third sector providers that adapted to working virtually reported that they planned to operate a hybrid model, continuing some remote delivery, which was found to be beneficial. A third sector organization working with individuals in HMYOI Polmont stated that by moving their services remotely by offering phone and digital support they were able to reach more people in need of support than they had been able to using a face to face approach.

## 4. Quantitative analysis to determine the scale of mental health need

### 4.1. Approach

Estimating the prevalence of mental health needs of Scotland's prison population can assist in planning service provision effectively in order to reduce the gap between health needs and interventions. Within the Marshall et al. (20) framework for health needs assessment in prison, data such as prison health surveys, routine service activity data provide helpful information which can be used to estimate the prevalence of mental health problems. However, there is no national, systematic process in place to comprehensively assess and monitor the level of mental health needs of those in Scotland's prisons. Additionally, due to COVID-19 restrictions, it was not possible to engage people currently in prison in screening or assessment for this study, and the brief project timescale coupled with ongoing service pressures for the NHS made it infeasible to gather and collate national data held by the NHS on routine service activity. In the absence of such direct data, quantitative analysis of existing secondary datasets were used to assist in estimating the proportion of people in Scotland's prisons who likely have a mental health problem. This is a valid alternative method to estimate prevalence of mental health need which is outlined in Marshall et al. (20).

#### 4.1.1. Scottish Health Survey

The Scottish Health Survey (SHeS) (42) is an annual survey conducted on the Scottish population in private households and is used and monitored as an indicator of health of the people in Scotland. The self-reported prevalence of certain common mental health problems is derived from this dataset, including anxiety, depression, alcohol use disorders and history of self-harm or suicide attempt. Data from SHeS were used to estimate an individual-level probability model for the non-prison population of Scotland having mental health needs. The 2019 dataset was used in the present study as it was the most recent year for which its methods were not substantively affected by the COVID-19 pandemic.

#### 4.1.2. Extract from the SPS PR2 system

SPS provided a dataset describing demographics of Scotland's prison population as of January 2022. These data were extracted from PR2, which is the operational information system used by SPS to manage the prison population. The PR2 variables used in this study were age, gender, ethnicity, and legal status.

#### 4.1.3. Forensic inpatient care

People with a mental illness, learning disability or related condition who are accused of or convicted of a criminal offense may be placed under the Criminal Procedure (Scotland) Act 1995, which allows the individual to be treated in hospital. Hospitals that accept these transfers include high, medium, low security forensic hospitals and intensive psychiatric care units. The Mental Welfare Commission (MWC) and the Forensic Network monitor the transfer of individuals from prison to psychiatric units under the Act. Data on prison-hospital transfers were retrieved from an MWC annual report (43) and from a report provided by the Forensic Network office.

### 4.2. Statistical analysis

The proportion of individuals in prison in Scotland who have mental health needs was modeled from available data on the non-prison population of Scotland. Individual likelihood of having one of five mental health problems was derived from the SHeS 2019 data using logistic regression and applied to the current prison population using the PR2 extracts. The five mental health problems modeled were:

- having a long-term mental health condition,
- having a history of deliberate self-harm or suicide attempt,
- drinking behavior consistent with a likely alcohol use disorder,
- anxiety symptoms in the previous week,
- and depression symptoms in the previous week.

Logistic regression was used to estimate the mental health needs of the prison population through modeling the mental health needs of the non-prison Scottish population. Demographic characteristics measured in both datasets were used as predictor variables: gender, age, ethnicity, and Scottish Index of Multiple Deprivation (SIMD) quintile.

Quantitative modeling occurred in a two-step process.

Step 1: The first step in this process was the estimation of the likelihood of having a mental health need based on individual demographics. The SHeS 2019 was used as it includes a nationally representative sample of individuals, both with and without mental health needs. Cases corresponding to individuals aged 16 years or older were retained for analysis. The following regression model was estimated using maximum likelihood estimation:


$$\begin{array}{r} has\text{\_}mental\text{\_}health\text{\_}need=\beta_{0}+\beta_{1}female_{i}\\ +\beta_{2}{ethnic\text{\_}minority}_{i}+\beta_{3}age_{i}+\beta_{4}deprivation_{i}+\varepsilon_{i} \end{array}$$

where:

- *i* represented each individual in the dataset,
- *has*_*mental*_*health*_*need* was a nominal dummy variable which takes the value of 0 if the individual does not have a mental health need and 1 if they do. A dummy variable was created for each of the five mental health needs modeled. The value of 1 was used according to the following criteria: the individual (1) reported having a long-term mental health condition; (2) reported a history of deliberate self-harm or attempted suicide; (3) scored 8 or higher on the AUDIT (44) indicating hazardous or harmful drinking; (4) reported two or more symptoms of depression in the previous week on the CIS-R (45) depression section; (5) reported two or more symptoms of anxiety in the previous week on the CIS-R anxiety section,
- *female* was a nominal dummy variable which took the value of 1 if the individual is female and 0 if the individual is male,
- *ethnic*_*minority* was a nominal dummy variable which takes on the value of 0 if the individual reported being white and 1 if the individual reported being from an ethnic minority group.
- *age* was an ordinal dummy variable indicating the individuals age in years according to specified bands: 16–20; 21–30; 31–40; 41–50; 51–60; 61–70; and over 70.
- *deprivation* was a dummy variable which takes on the value of 1 if the individual's SIMD is from the two most deprived quintiles, and a value of 0 if not.
- ε_*i*_ represented the error term corresponding to variance unaccounted for by the above predictor terms.

After estimating the equation, the probability of having each of the five mental health needs was predicted for each individual in the SHeS 2019 sample.

Step 2: In this step, the individual likelihood estimates derived from the SHeS 2019 sample were applied to every individual in Scotland's prison population, recreated using the PR2 extract. While the PR2 system does not hold information on the SIMD of the communities from which individuals come into prison, people in prison in Scotland are most likely to come from the bottom two SIMD quintiles (46). Therefore, in applying the likelihood estimates to the prison population, likelihood estimates corresponding to being in the bottom two SIMD quintiles were applied to the PR2 extracts.

After deriving probabilities for every individual based on age, gender, ethnicity, probabilities were then summed across different prison population subgroups to yield the proportion of the prison population who are likely to have a mental health need.

Descriptive statistics are reported relating to individuals in prison who require transfer to forensic inpatient facilities for assessment and treatment.

### 4.3. Results

#### 4.3.1. Prevalence estimates

Likelihood Ratio Chi-Square (*x*^2^) was significant for each model indicating improvement over the null model in each case.

- Long-term mental health condition: $x_{\left(9\right)}^{2}$ = 178.35, *p* < 0.001.
- History of deliberate self-harm or suicide attempt: $x_{\left(9\right)}^{2}$ = 54.24, *p* < 0.001.
- Alcohol use disorder: $x_{\left(9\right)}^{2}$ = 309.57, *p* < 0.001.
- Symptoms of anxiety: $x_{\left(9\right)}^{2}$ = 27.98, *p* = 0.001.
- Symptoms of depression: $x_{\left(9\right)}^{2}$ = 31.178, *p* < 0.001.

Quantitative modeling found that, relative to the mental health needs in the non-prison population, the estimated prevalence of all five mental health needs is higher for individuals in prison in Scotland. The estimated prevalence of mental health problems is set out in Table 2. The relative difference between the two populations was greatest for alcohol use disorders.

**Table 2:** Estimated prevalence [with 95% confidence interval (CI)] of mental health problems in Scotland's non-prison and prison population.

	**Scottish non-prison population (*****N*** = **4,903)**		**Scotland's prison population (*****N*** = **7,507)**	
	**%**	**95% CI**	**%**	**95% CI**
Long-term mental health condition	9.9	7.7–12.8	15.5	12.1–19.8
History of self-harm	9.5	5.5–16.1	17.0	10.0–27.3
Alcohol use disorder	14.1	11.3–17.5	29.9	24.9–35.9
Anxiety	12.1	7.4–19.2	16.0	9.6–25.6
Depression	10.9	6.5–17.9	17.9	10.7–28.7

#### 4.3.2. Use of forensic inpatient services

The Mental Welfare Commission for Scotland reports figures on the compulsory treatment of individuals subject to criminal proceedings. Assessment and treatment orders can be used to remand individuals to hospital for care. In 2018–2019, there were 222 assessment and treatment orders, 239 in 2019–20 and 204 in 2020–2021 (43). Orders of transfer for treatment direction (TTD) are used for the transfer of individuals who have been sentenced. According to the MWC there were 41 TTD orders issued in 2018–19, 36 in 2019–20 and 36 in 2020–21. Applying figures released by the Scottish Government (47) on the total number of sentenced individuals in custody each year, ~1% require inpatient psychiatric care in a given year (1.1% 2018–19; 1.2% 2019–20; data was not available for 2020–21).

The Forensic Network provided additional information on prison hospital transfers. Between 2018 and 2021, 20% of transfers were for women, although women make up only 3.6% of the current prison population. The majority of those transferred (62.3%) are on remand, even though people remanded to prison comprise 29.6% of the current prison population. The average number of days between date of referral and date of transfer ranged from 14.6 to 25.6 calendar days, an average of 21.1 days in 2021. The Department of Health and Social Care for England (48) recommends transfer take no more than 28 days from referral. There is no standard set out for Scotland.

According to the Forensic Network's comprehensive inpatient census undertaken on 26 November 2013 (the most recent data available), there were 111 patients in hospital who were admitted from prisons, comprising 21.3% of the forensic inpatient population at that time. The most represented diagnostic category among the people in prison who require forensic inpatient care is psychotic disorders (81.1%), the next largest group being affective disorders (5.4%), and personality disorder (4.5%).

## 5. Semi-structured interviews with professional stakeholders and individuals with lived experience

### 5.1. Approach

Qualitative semi-structured interviews were conducted to capture the perspective of professional stakeholders, individuals with experience of prison and mental health needs as well as their carers. Six interviews were conducted with community-based individuals with experience of prison and mental health needs; either their own experience or that of a carer. Contributions were also obtained from a group of three individuals transferred from prison to the high secure State Hospital, Carstairs for treatment. Six executive and senior-level stakeholders from SPS (with strategic, health, justice, and governance remits) were interviewed alongside representatives providing third sector, legislative, and welfare oversight. The operational perspective was sought from among nine SPS and NHS staff (two NHS consultants, Forensic Psychiatrist and Clinical Psychologist, two prison officers, 1 NHS health care manager, and four NHS mental health nurses) based within prisons and who had caring roles and responsibilities. Representation was obtained from establishments across the four prison monitoring regions, including sites that housed women, older adults and people on remand.

Topic guides were tailored to each group (professional stakeholders, lived experience participants, and carers), informed by published reports concerning mental health within prisons and reflected main aspects of the prison journey from reception to liberation. They broadly explored how mental health needs were assessed and supported across the prison journey including the provision of medication and access to resources within both the remand and sentenced environments. Stakeholders were also asked about staff attitudes, drug culture, the needs of specific groups, barriers to service provision, the implementation of previously made recommendations and what service improvements had been observed. Topic guides were assessed and approved by the RAG and Lived Experience panel. All interviews were conducted and recorded using Microsoft Teams, transcribed and imported to NVivo 12 Pro (49) for thematic analysis (50). Except where specified, all forms of mental health support, e.g., Psychological therapies, Occupational therapy, etc. are included within the concept of mental health support.

### 5.2. Results

#### 5.2.1. Perspectives of people in Scotland with lived experience of being in prison with mental health problems

##### 5.2.1.1. Reception, remand, and “jail life”

There was consensus among lived experience individuals that establishing immediate suicidal intent was the primary focus of mental health enquiry at reception into prison. Individuals felt highly stressed and “wracked with nerves” during reception and indicated it may be better to revisit some discussions a couple of days later. Those with multiple experiences of prison stated they were in “crisis mode” and thinking ahead to “jail life” issues such as “who's in prison? What have I got to worry about? Where am I going to get put? Who's going to be there? Have I got enemies and have I got friends... getting my stuff. Does my family know I've been moved prison?” They described how the responsibility was very much on the individual to engage and choose to share information with mental health services to gain any support.

Being housed within a remand hall presented a “chaotic,” “noisy,” and “volatile” environment. One person described being on remand as having “knocked me unwell.” Uncertainty in their living environment, with people constantly arriving and leaving along with no end in sight regarding criminal proceedings, led to a very “draining” experience for people, with little available to provide purposeful activity and distraction. In contrast, for some, remand was seen as a stable environment, providing a break from the stresses of living with homelessness and substance use problems.

##### 5.2.1.2. Relationships and interactions with officers and peers

Almost all individuals spoke of officer interactions in general terms that influenced how they expected officers to support their mental health needs. Day-to-day officer interactions shaped the development of trust and the extent to which they felt comfortable sharing mental health needs that are seen as a vulnerability in prison. Although individuals spoke of officers who “went above and beyond” providing or allowing “informal” mental health support, there was mention of those who “didn't give two monkeys.” Individuals indicated that they were unable to share mental health concerns with officers due to a general lack of “respect and dignity” they received from them, with a need for officers to recognize residents as “human beings” or that officers lacked training to provide appropriate support. Officers were viewed as gatekeepers who could deny access to mental health support and medication. Individuals did not feel listened to when they attempted to talk to officers.

There were also mixed opinions about sharing mental health needs with peers. These this included not trusting peers, concern about being labeled vulnerable and potentially exploited, alongside not wanting to burden others who have similar problems. There were mixed perceptions of a peer-support scheme called Listeners, which aims to reduce suicide and self-harm. Some saw Listeners as a valuable resource, others viewed it as a service that could be abused or something they would never engage with due to the Listener's position as a fellow resident and unable to affect change in their circumstances.

##### 5.2.1.3. Observation cells and the separation and reintegration unit (SRU)

Reinforcing a reluctance to share mental health needs with officers was a perception that “their answer to everything is throw you in a suicide cell. So, then you end up even more stressed because they put you in a daft outfit and then they put you on 15-min observations, even during the night.” It was noted that where officers did talk to residents there was an undertone of risk aversion “if you do this [die by suicide] it's on us.” The visibility of the observation cell next to the officer area was an additional reason given by individuals to lie about mental health needs even if questioned by officers. Placing someone in an observation cell has additional implications as the whole hall may need locked up to facilitate 15-min observations. Individuals described that this could lead to discord among peers, as could MH driven disruptive behaviors that disturb the whole hall.

Officers within the SRU were seen as more highly trained with a better understanding of mental illness than hall officers. The main negative aspect, which was also described in relation to observation cells, was that it was essentially an empty cell with nothing to distract from how they were feeling.

##### 5.2.1.4. Mental health needs, support, and coping strategies

Several individuals described how they made multiple disingenuous attempts to seek drugs from mental health teams to support substance use habits, or to sell for financial gain. Others admitted damaging their cells to convince doctors they required medication. In some cases, this behavior led to disrupted relationships with officers and mental health teams apparently denying access to mental health services when individuals were genuinely seeking support when they realized that their mental health was significantly deteriorating. Individuals described adopting coping strategies that helped them manage their own mental health including reading, listening to music, breathing techniques, and talking with members of the mental health team.

Many respondents with lived experience described having positive relationships with mental health teams. However, while they felt that being offered antidepressant medication seemed to be the answer to every mental health need, they also voiced a desire for talking therapies and for mental health staff to encourage greater engagement with available self-help resources, such as by demonstrating coping techniques like guided breathing.

Some individuals described that despite spending time in observation cells, including following attempted suicide, they had little contact with the mental health team. Family members voiced concern that the opportunity of stabilizing and addressing substance use problems or other drivers of mental ill health was not being utilized. In their view, attempts generally fail as engagement is central to mental health treatment within prisons yet many are unable to do so with a carer commenting that their partner “was too unwell to know to engage.” Family members also voiced that the needs of those with severe mental illness who avoid being placed in an observation cell or the SRU may be invisible to officers and therefore overlooked by the prison mental health team. This left families frustrated that missing the opportunity to address underlying needs would leave their loved ones repeatedly returning to prison.

##### 5.2.1.5. Liberation

Most individuals had experienced liberation at least once with little, if anything, positive said about the process. This included people being liberated after long-term sentences and from prisons individuals considered to be generally “good.” While liberation on parole was associated with greater throughcare planning regarding housing and benefit applications, little support for mental health and substance use problems was described except being told to see community teams, GP, etc. The lack of appropriate support after release, which contributed to disrupted transitions from custody to the community was viewed by individuals as a missed opportunity, particularly by family members. People gave multiple examples of being recalled to custody or being remanded within a few days of being released. Several described how they were released from prison with no support and found themselves homeless.

Successful transitions were reported when people received support from community psychiatric nurses and third sector in-reach work. Individuals described how engagement with third sector organizations, fostered by interaction with peer support workers, supported them through those first few high-risk weeks and helped break the imprisonment cycle by, for example, securing accommodation and therefore avoiding homelessness and the chaotic lifestyle that can bring.

#### 5.2.2. Executive and senior-level stakeholders

##### 5.2.2.1. Prison as a part of the wider justice system

Senior stakeholders commented it was difficult to reflect on mental health within the prison setting without considering it as an element of the wider justice system. Diversionary schemes that should be efficiently directing individuals from custodial disposal due to their evidenced needs were not perceived as operating efficiently.

##### 5.2.2.2. Scottish Prison Service corporate aims

Senior stakeholders recognized the impact of entering prison upon mental health and wellbeing. They also noted the corporate aims of SPS in relation to a role in identifying and supporting those with mental health needs. While SPS stakeholders acknowledged a focus on health within the prison service, they also mentioned the need for a more meaningful and joined up approach with greater strategic direction to overcome barriers. All senior stakeholders commented that improvements are being made, however further development was required with talk of the need for a “cultural shift” and that “a big sea change” was necessary for mental health to be more meaningfully supported within Scotland's prisons. It was commented that policy and practice needed to be more responsive to support the ever-changing needs of the prison population, for example the needs associated with looking after an aging population.

Most senior stakeholders discussed that underpinning this “cultural shift” was a focus on prisons adopting a more trauma-informed approach. Embracing a trauma-informed approach would place a greater emphasis on recovery within the prison environment and, in particular, the life journey that leads an individual to prison; for some on multiple occasions. While they recognized that prisons cannot “fix” everybody, their view was residents should leave prisons with better life opportunities than they arrived with. They noted that a lessening of the culture of risk aversion had led to a more person-centered approach within prisons. However, there were concerns surrounding the levels of scrutiny prisons are subject to, particularly where adverse events occur, such as a death in custody, and how that colors local decision-making in relation to mental health needs.

To reframe how prison officers care for individuals, most senior stakeholders mentioned a requirement for appropriate training, support, and resources to address the mental health issues facing officers on a daily basis and the development of a more trauma-informed environment. They indicated that the dynamic also requires change with officers engaging with residents rather than residents raising issues themselves. They indicated that relationships with partner agencies, such as third sector services, should be strengthened. It was voiced that both SPS and the NHS did not have a culture of sharing best practice or other knowledge exchange relating to service development.

##### 5.2.2.3. Prison as an extension of the community

Frustration was voiced that the prison environment is perceived as similar to the community when it comes to implementing recommendations or delivering health services. A failure to consider the legislative and risk management aspects associated with caring for an individual within prisons, and how that was reflected in day-to-day management was highlighted. A lack of recognition of how the physical environment and layout of prisons could impact upon the implementation of recommendations was also raised. Although it was expressed that there should be parity of access to services available in the community and within prisons, it was emphasized that they need to be delivered in a different way, for example by different staff groups or via virtual services. It was highlighted that community GP practices receive additional funds where they support patients from areas with high levels of multiple deprivation. Disparity in funding was noted as prisons do not receive those funds despite higher prevalence of demographic and social risk factors for mental health problems, and complexity and comorbidity among mental health needs of the prison population.

##### 5.2.2.4. Learning points from SPS's response to the COVID-19 pandemic

Concern was raised that access to mental health resources diminished during the height of the COVID-19 pandemic, primarily due to prison and NHS staff being required to cover essential services such as medication delivery. Residents who were already separating themselves from prison life due to mental health needs were also less visible to staff and could easily be overlooked.

Counterintuitively, positive feedback had been received from residents regarding being in small household bubbles with lock up at 5 pm and loss of evening recreation to limit viral spread through interpersonal mixing. Stakeholders described residents and officers reporting feeling a sense of safety through a reduction in mixing with others, better officer and resident interaction and the provision of mobile phones to facilitate in-cell communication with loved ones in the evening. SPS listened to feedback and indicated that a central tenant of prisons opening up after lockdown was that household bubbles and the associated sense of safety are maintained with a greater focus on providing meaningful activity to residents. It was highlighted, by a senior stakeholder, that the opportunity for staff and residents to get to know each other better within household bubbles led to improved, more trusting relationships and this could encourage residents to be more open about their needs with officers.

##### 5.2.2.5. Shared values, SPS/NHS alignment, and working relationship

It was generally recognized that the NHS and SPS have different corporate aims, and although they operate as partners, their relationship could be stronger. While there are difficulties for SPS in establishing consistency of approach across the nine NHS Boards that deliver services within prisons in Scotland, the NHS have similar challenges operating within prisons of different sizes leading to mental health teams operating differently. Senior stakeholders from both the NHS and SPS recognized the need for change to better support mental health needs within the prison environment. It was recognized that the COVID-19 pandemic had demonstrated that health was core to what SPS deliver: “If people don't feel well and feel safe and have got that emotional confidence that they can engage with people and with services, then we're not going to get very far.” Some prisons have established joint NHS/SPS partnership boards and they were able to act on published recommendations more readily.

Although most senior and some operational stakeholders spoke of good NHS/SPS relationships, there was a view that SPS and NHS should be communicating and working together more cooperatively to better support people living in prison. The overall impression was that the NHS and SPS did not always fully appreciate the extent of support they can provide one another.

#### 5.2.3. Executive, senior-level, and operational-level stakeholders

##### 5.2.3.1. Mental health needs of the prison population

Although there was little consistency reported in how mental health needs were detected by different prisons during the reception process and the days that followed, all methods involved various screening tools and members of both SPS and NHS staff. The one commonality was the need for the individual coming into prison to engage with staff and choose to share how they feel or what they are thinking at a point when they were likely to be feeling scared, uncertain or vulnerable.

Obtaining information about previous health treatment within the community and current prescription medications on reception involves a somewhat patchwork approach, with pockets of information available from various sources in a range of formats. It was highlighted that computer information systems and NHS Boards cannot always easily communicate with each other, posing significant issues of information sharing at entry and exit from custody.

There was uncertainty about whether there had been an increase in the number of residents presenting with mental health needs or if their mental health needs were simply being more readily identified and referred to services. There was, however, a shared perception that those being referred to mental health services were presenting with more complex needs. Underpinning this increase in the complexity of needs was the concept of trauma with residents either more comfortable with disclosing past trauma or staff more readily identifying trauma-related needs. Mental health services were striving to make officers more trauma-informed and formally/informally providing training and support around how to keep people safe whilst treating them in a compassionate, empathic, trauma-informed way. Instances were highlighted where officers were endeavoring to understand and support residents without automatically placing them on the formal suicide prevention strategy. While officers understood that for confidentiality reasons they were not privy to health information, they indicated that knowing a little more about residents would help them better understand behaviors and interact with those under their care as would more appropriate training and support.

Stakeholders felt that services are collectively failing people who have been to prison multiple times by not addressing past trauma and that they are simply “putting [a] sticking plaster over it,” and that “it feels like often it's firefighting.” This failing was related to a need for greater resources and training within both SPS and NHS.

##### 5.2.3.2. Resources and funding

Regarding resources, the overall picture was one of limitations relating to NHS staff shortages, the constraints of the physical environment within prisons and officer shortages, which affected service delivery and led to trained NHS staff underutilizing their skills covering non-role-specific tasks and delivery of medication. A clear view was that NHS staff were “under resourced and overworked” and that while there was a focus on mental health teams, this view extended across primary care and substance use services. Within prisons with only one mental health nurse, comment was made that their “caseload must be horrific.” However, another stakeholder from a better-resourced but small prison noted that the “luxury of being a small prison [is] we can spend more time with our patients.” These comments highlight the disparity across the prison estate in the number of residents cared for per WTE mental health nurse and the real-world impact that these differences make to resident care.

While an essential task, a majority of operational stakeholders noted that daily medication delivery takes a large amount of clinic time away from health care staff, with delivery highly dependent upon SPS regime. Individual prisons also operate different prescribing formularies with medications available within the community not always dispensed within prison.

NHS teams were creative in finding ways to adapt services to support the needs of their population within the available resources or address failures in recruitment and retention of staff. Operational stakeholders cited examples including making links with nursing courses and welcoming students on site. This served a dual purpose of raising the profile of nursing within the prison environment and providing extra support. Greater integration of substance use and mental health nursing teams helped provide a more wrap-around service to the exceptionally high numbers of residents with mental health and substance use issues. Advanced Nurse Practitioners have been recruited to support GPs with prescribing services. One service reported adopting a more community-orientated approach with all mental health referrals triaged through the GP service.

While NHS clinical psychology services have been developed at several prisons, not all have access. This disadvantages those in therapy who transfer to prisons without services. Despite limited staff and environmental resources, mental health teams are continually adapting and evolving to improve services, to meet their population needs and implement published recommendations.

More widely, there was a call for “more trained staff, be it officers or NHS staff, we need to understand more about it [mental health needs] before we can do anything about it.” Respondents explained that better mental health training for officers would reduce “inappropriate” referrals to mental health teams that are situationally driven and potentially transient rather than indicative of mental ill health. Appropriate training would also inform the development of a more trauma-informed environment and, along with the development of a directory of on-site and third sector service providers, support officers to signpost residents to services suitable to their needs.

##### 5.2.3.3. Observation cells/separation and reintegration units

There were mixed views from prison officer stakeholders about how often observation cells were used. One stated that they were regularly used to ensure the safety of an individual due to staff shortages. However, another officer noted observation cells being used only as a “last resort” and was unable to recall anyone in the recent past being placed on observation due to their mental health.

An executive/senior-level stakeholder questioned the effectiveness of placing those who express any degree of distress within an observation cell, devoid of interaction and stimulation and dressed in an anti-suicide smock. The further impact upon a person's mental health and potential future willingness to share distress was also questioned. Seeing people being placed into observation cells may, in and of itself, act as a barrier to others disclosing mental health concerns among the wider population. It was noted that there was no middle ground for those in mental distress between single bare cells and accommodation in large halls, with “safer” cells not always being the answer, although SPS were assessing observation cells and how they are used.

The perception among some senior-level stakeholders was that SRUs were increasingly utilized to house residents in extreme mental distress, although it was acknowledged that there can be difficulty in distinguishing behavior related to mental distress from violent and disruptive behavior. Where a lack of stimulation, peace and quiet were required, then the SRU was noted to provide that environment in comparison to the main hall. However, the use of SRUs and prison more generally as a place of safety was questioned, particularly for those in acute mental distress who require assessment for transfer to forensic hospital.

Concern regarding access to forensic psychiatric beds was raised. While high levels of staff input could be offered within the SRU this could also lead to difficulties reintegrating residents back to the main hall leading to resistive behaviors. Stakeholders cited regular discussions surrounding what support a resident required to transition from the SRU to the prison hall and, if they could not be delivered within the current establishment, then exploring transfer to another prison.

Stakeholders described using observation cells/SRU for the management of residents displaying psychotic symptoms related to use of novel psychoactive substances due to the risk they presented to themselves and others. The use of these drugs within Scotland's prisons was seen as inextricably linked to mental health needs and the underlying reasons for seeking and using substances.

##### 5.2.3.4. The needs of specific groups within the prison population

While recognizing that there were multiple specific groups within the prison population (for example, armed forces veterans, older adults, people with neurodevelopmental disorders), it was about “focusing on an individual and identifying what that person sees are their needs, rather than us [SPS/NHS] undertaking some sort of diagnosis or assessment. It's about that engagement.” However, in many cases interaction and management would be guided by NHS staff. Although NHS staff may be able to provide initial assessments and offer advice in relation to specific issues (for example, cognitive decline or alcohol-related brain damage), ideally specialist community services would link into the prison. There was a need for specialist services such as old age or substance use psychiatry, with some prisons in receipt of limited support, however funding was generally unavailable for specialist services. Links with third sector services were warmly mentioned and their contribution was widely recognized. Third sector services provided primarily support and assistance for substance use problems during liberation with separate groups operating to meet the specific needs of women. Third sector services had no formal links with health and wellbeing teams and were commonly linked to the recovery café/hubs operating within most prisons.

In general, those on remand had equal access to mental health services, although referral to psychological services, where available, could be restricted due to the short length of time people were expected to remain within prison. The availability of self-help resources and material that signposted residents to the mental health team was highlighted, in addition to the referral process which could be self-initiated, or through peers, or any staff member.

##### 5.2.3.5. Liberation

Executive stakeholders remarked that while third sector services provided support, there was a sense that it was an SPS responsibility to ensure a safe community transition and that all officers should be trained as Throughcare Support Officers. This could allow relationships built over time between residents and officers to be utilized, particularly for people serving longer sentences. While there were some good practices around liberation there was a lot more that could be done. Not every resident requires pre-liberation planning and neither was engagement with planning enforceable. NHS staff made links with mental health community teams where there was a need, set up appointments, shared information and provided a supply of some types of medication. There was, however, concern about the transition from custody to the community. It was recognized that the first few weeks of liberation could be challenging and chaotic. One mental health team member indicated they were attempting to standardize the liberation process while another noted that “the mental health and welfare [support] of our patients should cover people getting out.”

Half of executive stakeholders highlighted that liberation support appeared to fail for people on remand, who could often be liberated without warning. Individuals on remand could also leave prison late in the afternoon with no support or plan. Supporting those with the most complex needs through the liberation process was previously an SPS role, as staff knew the individual and their needs.

## 6. Discussion

The current study was part of a series of national needs assessments commissioned by the Scottish Government in 2020 in relation to Scotland's prison population to ensure that future changes to prison healthcare would be person-centered and evidence based. It was the first national assessment of the prison population's mental health needs since 2007 (26).

### 6.1. Key findings

The service mapping exercise found evidence of considerable variation in NHS service provision across Scotland's prisons. NHS staffing resources in prison did not appear to be closely linked with the size and characteristics of the prison population in individual establishments, which would be a parallel approach to how NHS Scotland resources are geographically allocated to individual NHS Boards (51). The observed and largely arbitrary variation is considered to lead to unintended inequalities leaving people who live in several prisons unfairly disadvantaged. Staffing vacancies, particularly among mental health nurses, appears to be a major barrier to meeting the mental health needs of individuals in prison. Beyond resourcing, service providers also highlighted wider challenges to supporting people in prison. They cited disruptions to care from mental health nurses being pulled away to support physical health and substance use services, problems in information sharing between professionals working in prisons, and constraints from prison facilities and the SPS regime on daily service delivery.

In the absence of robust indicators at the national level on the mental health needs of Scotland's prison population, the estimated prevalence of several mental health needs was modeled using data from Scotland's community population and fit to the prison population based on key demographic indicators. Analysis found that at least 15% of the prison population likely has a long-term mental health condition, 17% a history of self-harm, 30% an alcohol use disorder, 16% symptoms of anxiety, and 18% symptoms of depression in the past week. The derived mean prevalence estimates for each mental health problem was higher for all conditions relative to the non-prison population, consistent with known increased burden of mental health problems in people in prison (2). Data on the transfer of people from prison for inpatient psychiatric treatment between 2018 and 2021 indicated that, relative to Scotland's prison population as a whole, these individuals were disproportionately female or on remand, and a majority were transferred for the treatment of a psychotic disorder.

Interviews with professional stakeholders found there was a drive from the top of SPS to operate a more trauma-informed environment in Scotland's prisons. The COVID-19 pandemic had highlighted that the health and wellbeing of individuals in prison is foundational to the underlying aims of the prison service. Operationally, officers and NHS teams perceived residents as presenting with more complex mental health needs as well as trauma, and were striving to support residents with limited resources. From the resident perspective, the onus appeared to be very much on individuals to choose to engage and share information with prison mental health services to gain any support.

People with lived experience indicated that reception was a time of extreme stress and that beyond establishing acute needs (i.e., immediate suicidal intent), mental health needs should be explored more thoroughly a few days later. This group found being on remand to be a draining experience, characterized by uncertainty although for some it provided respite from homelessness. They acknowledged that some officers went above and beyond to support mental health needs, but the resident-officer dynamic needed improvement more generally. These participants found NHS mental health teams to be supportive when not operating under an excessive workload. Liberation was most successful where third sector and community services provided in-reach support ahead of someone being released and during the high risk first few weeks which could break the cycle of returning to prison, for example by securing housing.

### 6.2. Limitations

There are several limitations to the findings of this needs assessment resulting from the continuing COVID-19 pandemic. Face-to-face research was not possible during the timeframe of this project. This required taking an adapted approach using existing and secondary data and undertaking data collection through remote methods only. As the project was limited to use of secondary data, quantitative modeling was limited to use of fixed demographic variables as predictors of mental health needs, and could not include other relevant factors such as adverse life experiences and experiences related to imprisonment that increase the likelihood of having mental health needs. The prevalence estimates reported may therefore underestimate likely mental health needs. There were also several mental health needs including psychosis, personality disorder, and neurodevelopmental conditions, which were described in the literature review as experienced by people in prison in the UK, however the prevalence of these needs could not be estimated in this research due to the lack of available data. This report highlights the substantial service and workforce pressures experienced by those working to support people living in Scotland's prisons. Not all health professionals who wanted to engage with this needs assessment were able to due to pressures on clinical services and staffing problems exacerbated by the pandemic.

## 7. Conclusions

There is overwhelming evidence that individuals in prison are more likely to have a range of mental health needs, which are often multiple and complex. This study found that existing provision to support the mental health of people in prison in Scotland does not adequately meet these needs and that a change in approach is required.

Evidence from multiple elements of this needs assessment converged, indicating that a significant proportion of individuals in prison have, or will develop, mental health needs at some point in their journey. Our prevalence estimates were conservative, and taking into account the broader literature [reviewed in detail in the full report of this needs assessment (41)] people in prison are far more likely than not to have a mental health need. Like individuals in the community, the COVID-19 pandemic has likely had a negative impact on the mental health and wellbeing of Scotland's prison population. Recognizing this, and despite new challenges in service delivery resulting from the pandemic, many of which are still ongoing, this report found that the fundamental barriers to supporting the mental health of individuals in prison are likely longstanding. Professional stakeholders endorsed the view that individuals should leave prison better off and with greater opportunities than when they entered prison. To deliver this, however, there are substantial changes required in services delivered throughout the prison journey.

A mapping of current mental health service provision for people in and leaving prison highlighted that services in several establishments are insufficiently resourced. In those prisons, this equates to only an emergency service being provided, working with the most acutely ill, and leaving the majority of people in prison without support they could benefit from. NHS mental health teams are under-resourced and overworked, attempting to innovatively manage their workloads as effectively as they can within their limited resources. While acknowledging these challenges, it should be highlighted that there have been major positive developments in both the overall size and multi-disciplinary composition of prison mental health teams in Scotland since the last previous national mapping exercise in 2012. Nearly all prisons now have formal input from mental health nursing, psychiatry, and clinical psychology and ~½ have AHPs as part of the mental health team. Compared to the previous mapping exercise, input from clinical psychology and AHPs has increased considerably. Expansion of the mental health MDT increases access to appropriate care for more people, including some with mild mental health problems. These developments are welcomed.

Unfortunately, there are fundamental issues with attracting and retaining staff to work in prisons against the backdrop of high demand for services. Staff absences brought on by the pandemic have further exacerbated resource pressures. Professionals highlighted a number of challenges to meeting mental health needs of people in prison, but a common theme was observed in relation to difficulties in and barriers to coordinated and joint working across SPS, health and third sector organizations to support individuals in prison. According to Scotland's prison health promotion framework, all who work in prisons bear a duty to support the mental health and wellbeing of people in prison, and there are corresponding roles for all agencies in implementing the necessary actions to do so.

Several reports published in the last decade have highlighted concern around many of the same problems identified in this report and offered appropriate, evidence-based recommendations to address them (33, 52). Despite repeated scrutiny of the same issues, most recommendations have not been fully implemented. This suggests that current structures and operational arrangements do not facilitate the development of innovative practice or are too restrictive to enable the change required. A fundamental change to prison mental health services in Scotland is required.

### 7.1. High-level recommendations

Following on from the findings of this needs assessment, a series of evidence-based recommendations were developed. These are intended to address a range of issues identified by this study, from high-level, strategic issues to daily operations including resourcing and service delivery. Implementing these recommendations will require action and in many instances, coordinated action from multiple actors including the Scottish Government, NHS Scotland, the Scottish Prison Service, local authorities, and third sector organizations as relevant. Six high-level recommendations are listed below as they may resonate with professionals and researchers around the issues facing local prison mental health care in other countries. The remaining 12 recommendations are more straightforward solutions to operational issues, and likely to be more specific to the local Scottish context and service arrangements. These can be reviewed in the full published report (41).

#### 7.1.1. Recommendation 1

A fundamental change is required in how the mental health of individuals in prison is perceived, given the demonstrated mental health needs of Scotland's prison population. A model of care should be adopted across all prisons that focuses on assessing and meeting individual needs, supporting individuals' wellbeing, and providing a caring and supportive environment. Trauma-informed care is one model that may be appropriately considered.

#### 7.1.2. Recommendation 2

The model of care adopted should have individuals' needs and wellbeing at its center and strive to make the prison environment more therapeutic with a greater focus on meaningful activity. To break the cycle of repeated imprisonment, individuals should leave Scotland's prisons with better life opportunities than when they started their sentence.

#### 7.1.3. Recommendation 3

Greater resources are required for NHS mental health services. Rather than use community-based formulations, modeling should be used to determine service provision, accounting for the known demographic and social characteristics of the population in each prison, recognizing that most individuals come from communities of multiple deprivation, have had adverse life experiences and many have multiple and complex needs. The outcomes of these models for each prison should be published.

#### 7.1.4. Recommendation 4

An increase in funding for clinical psychology and allied health professionals within the multidisciplinary mental health team is needed in many of Scotland's prisons where current input is either none or limited. As the model of care is developed, a need for increased resources from other professional groups may too become apparent.

#### 7.1.5. Recommendation 5

Standards for prison mental healthcare should be adopted. These could be newly developed or adopted from existing standards such as those published by the Royal College of Psychiatrists (53). Adopted standards should include staffing requirements per prison resident to ensure consistency across the estate.

#### 7.1.6. Recommendation 6

The development of a formal partnership between SPS (and private contractors), health and social care, and third sector organizations is necessary to drive forward the high-level changes recommended. This partnership should be empowered to deal with strategic and operational issues across the prison and health services. This must include a mechanism to empower decision making across all NHS Boards that interface with the prison estate. There should be mechanisms for governance, and processes embedded to enable routine quality improvement and assurance.

## Data availability statement

The raw data supporting the conclusions of this article will be made available by the authors, without undue reservation.

## Ethics statement

Ethical review and approval was not required for the study on human participants in accordance with the local legislation and institutional requirements. Written informed consent for participation was not required for this study in accordance with national legislation and institutional requirements. However, for good practice written informed consent was received from lived experience participants.

## Author contributions

LG, CR, SH, and LT designed the study and wrote the protocol. SH provided quality assurance role between the study team and the funder, Scottish Government. LG, CR, and CK designed data collection tools used and undertook data collection. LG and CR undertook statistical and qualitative analysis, respectively, and wrote the first draft of the manuscript. All authors commented on the manuscript and contributed to and have approved the final manuscript.

## Footnotes

^1^This manuscript uses the term “mental health needs” to refer to the broad set of psychological and behavioral problems associated with mental disorder, personality disorder, substance use, neurodevelopmental disorder and other brain conditions for which mental health services can offer support.

^2^From the authors' perspectives, these apparently arbitrary differences in mental health team staffing across Scottish prisons may, to an extent, be driven by two factors. The first is local health board response to public inquiries following adverse incidents or inspection visits by the Mental Welfare Commission for Scotland. A second is the effect of having senior forensic mental health professionals employed in a health board area or specific prison establishments who is particularly supportive of expansion to the multidisciplinary mental health in-reach team.
